# Navigating the Landscape of Hydrocortisone Administration in Septic Shock: Current Concepts and Future Directions

**DOI:** 10.7759/cureus.49870

**Published:** 2023-12-03

**Authors:** Anirudh Kommareddy, Jayant D Vagha, Revat J Meshram

**Affiliations:** 1 Pediatrics, Jawaharlal Nehru Medical College, Datta Meghe Institute of Higher Education and Research, Wardha, IND

**Keywords:** precision medicine, evidence-based practice, inflammatory response, critical care, hydrocortisone, septic shock

## Abstract

Sepsis remains a formidable challenge in critical care medicine, often culminating in a life-threatening condition known as septic shock. This review article navigates the intricate landscape of hydrocortisone administration in septic shock management, delving into historical perspectives, current evidence, controversies, mechanisms of action, practical considerations, and the importance of precision medicine. Hydrocortisone's role as an adjunctive therapy is explored, highlighting its potential to stabilize hemodynamics, mitigate the inflammatory response, and improve patient outcomes. However, debates persist regarding patient selection, dosing regimens, safety profiles, and long-term consequences. The future of septic shock management lies in emerging therapies, precision medicine approaches, biomarker discovery, and targeted interventions. Moving forward, exploring novel therapeutic avenues, understanding patient-specific responses, and uncovering potential biomarkers will be crucial in advancing septic shock treatment strategies. Clinical guidelines provide a foundation, but individualized patient care, interdisciplinary collaboration, and ongoing research are essential to optimize treatment strategies. This article underscores the call for continued research and evidence-based practice as we strive to enhance the care of septic shock patients and pursue improved outcomes in this critical condition. Embracing future developments in the field will enable us to adapt and refine our approach, ultimately contributing to the advancement of septic shock management.

## Introduction and background

Sepsis remains a formidable challenge in modern medicine, with septic shock being one of its most critical and life-threatening manifestations. Septic shock is characterized by a dysregulated host response to infection, leading to systemic inflammation, widespread tissue damage, and organ failure. This condition carries an alarmingly high mortality rate, making it a focal point of research and clinical interventions in critical care settings [1-3].

Septic shock's clinical picture is defined by a constellation of symptoms, including profound hypotension, impaired tissue perfusion, and organ dysfunction, often resulting in multi-organ failure. The pathophysiological underpinnings of septic shock involve a complex interplay between the invading pathogen, the host's immune response, and various systemic factors. These intricate mechanisms demand a comprehensive understanding to develop effective therapeutic strategies [1,2].

One of the critical elements in the management of septic shock is the administration of hydrocortisone, a glucocorticoid hormone. The use of hydrocortisone in septic shock management has sparked significant interest, debate, and research over the years. This intervention is grounded in the premise that septic shock patients may experience relative adrenal insufficiency, leading to inadequate cortisol production amid their overwhelming stress response [4].

Hydrocortisone, a synthetic form of cortisol, has been postulated to address this potential deficiency by providing anti-inflammatory and immunomodulatory effects. These effects aim to counteract the uncontrolled systemic inflammation observed in septic shock, stabilizing the patient's hemodynamics and improving their overall clinical outcome. However, the administration of hydrocortisone has its controversies, and the selection of appropriate patients and dosing regimens remains a subject of ongoing investigation [5].

This comprehensive review article explores the intricate landscape of hydrocortisone administration in septic shock. By synthesizing the existing literature, we aim to provide a comprehensive overview of the current concepts, controversies, and emerging directions related to hydrocortisone therapy in septic shock management.

## Review

Methodology

Conducting a comprehensive and well-planned search strategy was of utmost importance. The search strategy comprised various essential components, commencing with selecting appropriate databases to guarantee thorough coverage of the pertinent literature. Databases such as PubMed/MEDLINE, Embase, Cochrane Library, Scopus, Web of Science, CINAHL, and Google Scholar played pivotal roles in identifying scholarly articles and research findings. To retrieve relevant articles effectively, a meticulously curated set of keywords and Medical Subject Headings (MeSH) terms was employed. These search terms encompassed crucial concepts, including "septic shock," "hydrocortisone," "corticosteroids," "clinical trials," "patient selection," and "safety considerations," among others. Boolean operators and truncation symbols allowed for combining and expanding search terms as needed. Furthermore, clear inclusion and exclusion criteria were established to ensure alignment with the review's objectives. This entailed including clinical trials, systematic reviews, and meta-analyses published in peer-reviewed journals. Simultaneously, studies with methodological deficiencies or those unrelated to septic shock and hydrocortisone were excluded from consideration. In addition to these measures, the review also considered the potential value of gray literature, encompassing conference materials, theses, and reports. Recognizing that gray literature can provide unique insights not typically found in traditional peer-reviewed journals, it was considered part of the search strategy. This diligently crafted search strategy facilitated a comprehensive exploration of hydrocortisone administration in septic shock, ensuring the review article was grounded in a well-rounded assessment of the available literature. This approach aimed to provide an informative and up-to-date review of the topic.

Pathophysiology of septic shock

Inflammatory Response in Septic Shock

Septic shock is characterized by a profound and dysregulated inflammatory response to infection. When pathogens like bacteria, fungi, or viruses enter the bloodstream, they trigger an immediate immune response. This immune response involves the release of proinflammatory cytokines, such as interleukin-1 (IL-1), interleukin-6 (IL-6), and tumor necrosis factor-alpha (TNF-α), by immune cells. These cytokines play a crucial role in recruiting immune cells to the site of infection, enhancing phagocytosis, and activating the complement system [6]. However, in septic shock, this inflammatory response becomes excessive and uncontrolled. The immune system's regulatory mechanisms fail, leading to a "cytokine storm." This cytokine storm contributes to systemic inflammation, vasodilation, increased vascular permeability, and the activation of coagulation pathways. As a result, blood pressure drops, and vital organs suffer from inadequate perfusion, leading to organ dysfunction and failure [7].

Role of Cortisol in Stress Response

Anti-inflammatory effect: Cortisol is renowned for its potent anti-inflammatory properties. In the face of stress, whether caused by infection or injury, the immune system often initiates a robust inflammatory response as a defense mechanism. While this response is essential for fighting off pathogens and promoting tissue repair, it can become excessive and harmful. Cortisol steps in to maintain a delicate balance. It suppresses the production of proinflammatory cytokines, signaling molecules that drive inflammation. By doing so, cortisol helps prevent an exaggerated and damaging immune response that can lead to tissue destruction and organ dysfunction [8].

Metabolic effects: Another vital role of cortisol is regulating glucose metabolism during stressful situations. Cortisol works to increase blood glucose levels through several mechanisms. Firstly, it promotes gluconeogenesis, a process where glucose is synthesized from non-carbohydrate sources, such as amino and fatty acids. This ensures that a readily available energy source is circulating in the bloodstream. Additionally, cortisol decreases glucose uptake by peripheral tissues, such as muscle and fat cells. This effect helps preserve glucose for critical functions, such as powering the brain and fueling vital organs when the body is under stress [9].

Vascular tone regulation: Cortisol also modulates vascular tone, which refers to the constriction or relaxation of blood vessels. In stressful situations, maintaining adequate blood pressure is essential to ensure a sufficient supply of oxygen and nutrients to vital organs, particularly the heart and brain. Cortisol influences vascular tone by altering the sensitivity of blood vessels to various regulatory factors, including vasoconstrictors (agents that narrow blood vessels) and vasodilators (agents that widen blood vessels). By regulating vascular tone, cortisol helps maintain blood pressure within a range that supports overall physiological function during stress [10].

Dysregulation of Cortisol in Septic Shock

In septic shock, the stress response becomes dysregulated. Some patients with septic shock may experience relative adrenal insufficiency, which means their adrenal glands do not produce enough cortisol to meet the increased demand caused by the severe stress of infection. This dysregulation can be due to several factors, including the direct effects of inflammation on the adrenal glands and alterations in the hypothalamic-pituitary-adrenal (HPA) axis [11]. The consequences of dysregulated cortisol production in septic shock are significant. Insufficient cortisol levels can exacerbate the inflammatory response, leading to a more severe cytokine storm and worsening vasodilation and vascular permeability. This, in turn, can contribute to the hemodynamic instability and organ dysfunction characteristic of septic shock [12].

Historical perspective on hydrocortisone use in septic shock

Early Studies and Controversies

Pioneering investigations: The genesis of corticosteroid therapy in septic shock management can be traced back to the 1950s and 1960s. These pioneering studies instilled hope regarding the potential benefits of using corticosteroids in this critical condition. Initial trials suggested that corticosteroid administration could lead to several positive outcomes, including improving hemodynamics, stabilizing blood pressure, and overall patient well-being. These early investigations kindled enthusiasm for the therapeutic potential of corticosteroids as a tool in septic shock management [13].

Controversial findings: While the initial studies offered promise, subsequent investigations in the following decades introduced a contentious dimension to using corticosteroids in septic shock. These later trials often produced conflicting results, injecting uncertainty into the medical community's perception of corticosteroid therapy. Skeptics of corticosteroid use pointed to potential risks of this treatment approach, such as immunosuppression and elevated susceptibility to secondary infections. These concerns led to vigorous debates and a lack of consensus on the role of corticosteroids in septic shock management [13].

The controversies surrounding early studies and their subsequent findings underscore the complexities inherent in septic shock treatment. The variability in study outcomes and potential adverse effects necessitated a nuanced examination of corticosteroid therapy in this context. Subsequent research and evolving guidelines have sought to provide greater clarity regarding the role of hydrocortisone and other corticosteroids in septic shock, contributing to a more informed and cautious approach to their use in contemporary medical practice [14].

Evolution of Guidelines and Recommendations

Consensus conferences: In the late 1990s and early 2000s, the emergence of international consensus conferences, most notably the Surviving Sepsis Campaign, marked a pivotal juncture in addressing the role of corticosteroids in septic shock management. These conferences convened experts worldwide to deliberate on the best practices for sepsis and septic shock care. They played a critical role in shaping guidelines and recommendations by providing a platform for synthesizing available evidence and establishing consensus among experts [1].

Guideline updates: Subsequent iterations of sepsis and septic shock guidelines, influenced by the deliberations of consensus conferences and the accumulation of clinical data, gradually incorporated hydrocortisone therapy into their recommendations. These guidelines provided specific criteria for identifying patients who might benefit from corticosteroid treatment. Typically, this included patients with refractory hypotension despite adequate fluid resuscitation and vasopressor support. Including hydrocortisone in these guidelines represented a shift in the management paradigm, acknowledging the potential value of corticosteroids in specific subsets of septic shock patients [1].

Fluidity of recommendations: It is crucial to recognize that the recommendations concerning hydrocortisone use in septic shock have remained stable. They have evolved and adapted in response to emerging evidence, ongoing research, and a deeper comprehension of the complexities of critical care medicine. This fluidity reflects the commitment of the medical community to evidence-based practice and its readiness to refine guidelines to optimize patient care [15].

Critiques of Historical Research

Methodological limitations: Early studies investigating the use of hydrocortisone in septic shock often faced substantial methodological limitations. These studies were frequently plagued by small sample sizes, heterogeneity in patient populations, and a need for standardized protocols. Such limitations make it challenging to draw definitive conclusions about the efficacy and safety of hydrocortisone. The variability in study designs and patient characteristics has hindered the ability to extrapolate findings to broader septic shock populations [16].

Publication bias: Publication bias is a concern in medical research, where studies with positive or statistically significant findings are more likely to be published than those with negative or inconclusive results. Regarding hydrocortisone therapy for septic shock, publication bias can skew the perception of its effectiveness. If positive results are overrepresented in the literature, it can create an overly optimistic view of the treatment's benefits [17].

Changing paradigms: As our understanding of septic shock's pathophysiology has advanced, so too have the critiques of historical research. Critics argue that the benefits and risks of corticosteroid therapy must be considered within the context of each patient's specific immune and inflammatory profile. Septic shock is increasingly recognized as a heterogeneous condition with varying underlying mechanisms. Consequently, the response to hydrocortisone may differ among patients with distinct pathophysiological subtypes. The one-size-fits-all approach of historical studies may need to account for this heterogeneity adequately [18].

Current evidence on hydrocortisone administration in septic shock

Clinical Trials and Systematic Reviews

Large-scale clinical trials have significantly shaped the research landscape surrounding hydrocortisone therapy in septic shock. These trials have played a crucial role in expanding our understanding of how hydrocortisone impacts patient outcomes in this critical condition. Two notable examples that have contributed substantially to our knowledge base are the HYPRESS (Hydrocortisone for Prevention of Septic Shock) trial and the ADRENAL (Adjunctive Corticosteroid Treatment in Critically Ill Patients with Septic Shock) trial. Both trials have enrolled substantial septic shock patients, yielding robust datasets for comprehensive analysis [19].

The HYPRESS trial was designed to explore the prophylactic use of hydrocortisone in patients at risk of developing septic shock. This groundbreaking study sought to determine whether early hydrocortisone administration could prevent the progression to full-blown septic shock, potentially altering the disease trajectory for these patients [19].

In contrast, the ADRENAL trial focused on using hydrocortisone as an adjunctive treatment in patients who already had established septic shock. This trial investigated whether hydrocortisone could improve critical outcomes, including mortality rates and shock duration, among patients in this particular population [20].

Beyond individual trials, systematic reviews and meta-analyses have played a pivotal role in synthesizing and interpreting the collective evidence on hydrocortisone therapy in septic shock. These rigorous analyses combine data from multiple clinical trials to comprehensively overview the cumulative evidence. Doing so provides a clearer perspective on the overall effect size of hydrocortisone therapy and identifies any consistent trends or patterns across different studies [21].

Systematic reviews and meta-analyses are instrumental in identifying the cumulative impact of hydrocortisone therapy. They can help elucidate whether hydrocortisone consistently demonstrates beneficial effects on important outcomes such as mortality, hemodynamics, or other relevant factors [22]. Moreover, these analyses can delve into the nuances of patient populations, exploring whether specific subgroups derive more significant benefits from hydrocortisone therapy. This personalized approach to treatment consideration aids in the refinement of patient selection criteria and provides valuable guidance for clinical decision-making [23].

Efficacy of Hydrocortisone in Improving Outcomes

Hemodynamic stabilization: Hydrocortisone consistently demonstrates its ability to stabilize hemodynamics in septic shock patients. A pivotal effect of hydrocortisone is the reduction of vasopressor requirements, a phenomenon particularly evident in patients with relative adrenal insufficiency (RAI). This subset of septic shock patients often presents with impaired cortisol production and an inadequate physiological response to stress [24]. Hydrocortisone's intervention in such cases plays a vital role in restoring vascular tone and normalizing blood pressure, contributing to the patient's overall stability.

Vasopressor reduction: The administration of hydrocortisone frequently decreases the dosage and duration of vasopressor medications needed to maintain blood pressure within the desired range. This outcome is facilitated by hydrocortisone's ability to promote vascular tone and enhance vascular responsiveness, facilitating more effective blood pressure control. Improved blood pressure control, in turn, supports vital organ perfusion [25].

Mortality reduction: The impact of hydrocortisone on mortality rates among septic shock patients remains a subject of ongoing debate and further investigation. While findings may exhibit variability across studies, certain trials, and systematic reviews have reported reduced mortality rates among specific subgroups of septic shock patients receiving hydrocortisone [16]. This observation underscores the nuanced and potentially lifesaving role that hydrocortisone can play in specific clinical contexts.

Subgroup benefits: Subpopulations, particularly those with documented relative adrenal insufficiency, appear to derive more substantial mortality reduction benefits from hydrocortisone therapy. Identifying and targeting these high-risk individuals may enhance the potential benefits of corticosteroid treatment, emphasizing the importance of patient stratification in clinical decision-making [26].

Time to shock resolution: Hydrocortisone therapy may contribute to an expedited resolution of shock in septic shock patients. This accelerated resolution can lead to a quicker weaning of vasopressors and improved organ perfusion [4]. The ability to expedite shock resolution is critical in managing septic shock, as it helps mitigate the risk of organ damage and failure.

Faster weaning: Hydrocortisone's capacity to reduce or discontinue vasopressor support is pivotal in septic shock management. The rapid response to therapy facilitates the weaning process, alleviating the burden on the cardiovascular system and reducing the potential complications associated with prolonged vasopressor use [27]. This aspect underscores the practical implications of hydrocortisone therapy in clinical practice.

Patient Selection Criteria

In determining eligibility for hydrocortisone therapy in cases of septic shock, a crucial factor to consider is the presence of adrenal insufficiency. Identifying patients who exhibit relative adrenal insufficiency (RAI) is paramount because they will likely benefit most from hydrocortisone treatment. RAI is characterized by impaired cortisol production in response to stress, resulting in an inadequate physiological response [28].

Adrenal insufficiency testing: The cornerstone for diagnosing adrenal insufficiency is corticotropin (ACTH) stimulation tests. These tests evaluate the adrenal gland's ability to produce cortisol when exposed to ACTH administration. An insufficient cortisol response during these tests serves as a clear indicator of adrenal insufficiency [29].

Alternative biomarkers: In certain circumstances where corticotropin stimulation tests may not be feasible or readily accessible, alternative biomarkers, such as serum cortisol levels or free cortisol indices, can also prove valuable in identifying patients showing signs of RAI [30]. The severity of septic shock and the patient's response to initial resuscitation efforts should assume a pivotal role in the decision-making process concerning hydrocortisone therapy [31]. Patient selection criteria may encompass the following considerations:

Refractory shock: Hydrocortisone is frequently contemplated in cases of refractory septic shock, wherein patients fail to attain adequate blood pressure control despite undergoing fluid resuscitation and receiving vasopressor support. Refractory shock implies a deficiency in the effectiveness of conventional interventions, and hydrocortisone may be introduced as an adjunct to enhance hemodynamic stability [32].

Persistent hypotension: Patients who consistently exhibit hypotension despite implementing appropriate resuscitation efforts may also qualify as candidates for hydrocortisone therapy. This persistent hypotension may indicate an insufficient stress response, and the administration of hydrocortisone can assist in bolstering vascular tone and achieving better control over blood pressure [33].

Dosing Regimens and Duration of Treatment

Dosing strategies in hydrocortisone administration for septic shock have been explored in clinical trials and practice. Two primary dosing approaches have been considered: continuous infusion and bolus dosing. The choice between these strategies should be guided by clinical judgment and tailored to the patient's needs. Continuous hydrocortisone infusion offers the advantage of maintaining a more stable and sustained cortisol level in the bloodstream. This continuous approach proves particularly beneficial for patients with severe septic shock necessitating constant hemodynamic support, as it minimizes fluctuations in cortisol levels commonly associated with bolus dosing [34]. On the other hand, bolus dosing may be more appropriate in situations where rapid hemodynamic stabilization is imperative, such as the initial phase of treatment. Bolus doses are administered as intravenous injections, typically over a brief period, to achieve a more immediate therapeutic effect. This bolus dosing approach can be employed independently or with continuous infusion based on ongoing clinical assessments [35].

Regarding the duration of hydrocortisone therapy in septic shock, it remains a topic of ongoing debate and research. While certain studies have suggested that shorter courses of hydrocortisone treatment may be as effective as longer ones, this area requires further investigation and clarification. Key considerations about the duration of treatment encompass adopting a tailored approach based on individual patient responses, underlying adrenal function, and the clinical course of the septic shock. In some instances, a brief course of hydrocortisone therapy may suffice to attain hemodynamic stability, while in others, a more extended duration may be warranted [36]. Moreover, conducting a comprehensive risk-benefit assessment is crucial when determining the duration of hydrocortisone therapy. This assessment should consider potential risks associated with prolonged corticosteroid use, such as the heightened susceptibility to secondary infections and the possibility of adrenal suppression. Vigilant monitoring of patients throughout and following treatment is essential to promptly detect any adverse effects and ensure their well-being [37]. As the landscape of evidence in this field continues to evolve with insights from ongoing clinical trials and research, guidelines and recommendations concerning the optimal hydrocortisone therapy duration may be revised. Thus, clinicians must remain abreast of the latest findings and be prepared to adapt their clinical practices accordingly [38].

Safety Considerations

Several safety concerns are associated with corticosteroid therapy, including using hydrocortisone in septic shock management. One significant concern is the heightened risk of secondary infections, as corticosteroids can suppress the immune system, potentially compromising the body's ability to combat pathogens effectively [39]. Precise patient selection is crucial to mitigate this risk, involving identifying individuals at higher risk of infection-related complications and those displaying signs of relative adrenal insufficiency. Close monitoring for signs of new or worsening infections throughout hydrocortisone therapy is imperative for patient safety and optimal outcomes [40].

Another concern is the potential for hyperglycemia, particularly among patients with preexisting diabetes or impaired glucose metabolism. Hydrocortisone administration can lead to elevated blood glucose levels through increased gluconeogenesis in the liver and reduced glucose uptake by peripheral tissues [41]. Consequently, continuous glucose monitoring and diligent insulin management may be necessary, especially for patients with a history of diabetes. Close monitoring and adjusting insulin therapy can help mitigate the risk of uncontrolled hyperglycemia [42].

Moreover, corticosteroids, including hydrocortisone, may induce psychological and neuromuscular side effects such as mood changes, agitation, insomnia, and muscle weakness. Although these effects are typically associated with long-term or high-dose corticosteroid use, they should be monitored during treatment to ensure patient well-being [43]. Comprehensive patient assessment, including the evaluation of psychological well-being and neuromuscular function, is essential for maintaining patient safety and addressing any observed mood or muscle strength changes as needed [44].

Mechanisms of action

Anti-inflammatory Effects

Suppression of proinflammatory cytokines: One of the hallmark actions of hydrocortisone is its ability to suppress the production of proinflammatory cytokines. In septic shock, there is often an excessive release of cytokines such as interleukin-1 (IL-1), interleukin-6 (IL-6), and tumor necrosis factor-alpha (TNF-α). These cytokines are central drivers of the systemic inflammatory response, contributing to the characteristic symptoms of shock, organ dysfunction, and tissue damage. Hydrocortisone intervenes in this process by downregulating the synthesis and release of these cytokines. Doing so helps restore the balance of the immune response and prevents the uncontrolled amplification of inflammation [45].

Inhibition of immune cell activation: Corticosteroids, including hydrocortisone, exert their anti-inflammatory effects by inhibiting the activation and migration of immune cells such as macrophages and neutrophils. In septic shock, these immune cells are activated in response to invading pathogens, releasing inflammatory mediators and infiltrating tissues. While this immune response is essential for pathogen clearance, it can also lead to collateral tissue damage. Hydrocortisone moderates this immune cell response, reducing their infiltration into vital organs and dampening the extent of tissue injury. This helps preserve organ function and contributes to a more controlled and regulated immune response [46].

Modulation of eicosanoid pathways: Hydrocortisone further exerts anti-inflammatory influence by modulating eicosanoid pathways. Eicosanoids, such as prostaglandins and leukotrienes, are lipid mediators initiating and propagating inflammation. Corticosteroids like hydrocortisone reduce the production of these pro-inflammatory eicosanoids, diminishing their impact on the inflammatory cascade. By doing so, hydrocortisone helps prevent excessive vasodilation, increased vascular permeability, and tissue edema associated with uncontrolled eicosanoid release [47].

Hemodynamic Effects

Vascular tone regulation: Within the intricate landscape of blood pressure regulation, cortisol, the biologically active hydrocortisone, wields significant influence over vascular tone. This facet becomes particularly salient in septic shock, characterized by a disordered inflammatory response that often culminates in widespread vasodilation and hypotension. Cortisol's impact on blood vessels takes center stage in this scenario, holding promise as a key modulator of vascular dynamics [48].

Enhanced vasoconstriction: A noteworthy facet of cortisol's role lies in its ability to augment the sensitivity of blood vessels to vasoconstrictors like norepinephrine. This signifies that cortisol potentiates its effects when vasoconstrictive agents are administered to elevate blood pressure. Consequently, cortisol contributes significantly to vasoconstriction, countering the excessive vasodilation frequently observed in septic shock. This pivotal function is instrumental in maintaining appropriate blood pressure and tissue perfusion [49].

Reduced sensitivity to vasodilators: In addition to enhancing vasoconstrictor effects, cortisol also diminishes the responsiveness of blood vessels to vasodilatory stimuli. This dual action underscores its pivotal role in upholding vascular tone. By attenuating the response to vasodilator signals, cortisol is a bulwark against the potentially deleterious decline in blood pressure that often manifests in the throes of septic shock [50].

Improvement in microcirculation: Hydrocortisone has demonstrated a noteworthy capacity to ameliorate microcirculatory blood flow in patients grappling with septic shock. This phenomenon bears profound implications for tissue oxygenation and organ function, as it nurtures the vitality of the microcirculation, a system critical to cellular oxygen delivery and waste removal [51].

Enhanced microcirculation: Sepsis and septic shock cast a shadow on microcirculatory perfusion, potentially leading to insufficient oxygen delivery to vital organs and, by extension, contributing to organ dysfunction. Hydrocortisone's prowess in enhancing microcirculatory blood flow serves as a salutary response to this issue, promoting the optimization of capillary and small vessel perfusion. This heightened microcirculation translates into more efficient oxygen exchange at the tissue level, potentially mitigating the specter of organ damage in the context of septic shock [52].

Effects on Immune Function

Corticosteroids, including hydrocortisone, influence the immune system by performing immunosuppressive actions. These actions inhibit critical immune cell functions, notably affecting T and B cells. While this immunosuppressive capability can prove beneficial in reigning in excessive inflammation and alleviating the adverse consequences of an overactive immune response, it also raises concerns about the potential for heightened vulnerability to secondary infections [53]. One of the primary targets of corticosteroid interference is the hindrance of immune cell activation and function, with a particular focus on T lymphocytes. These T cells are pivotal in orchestrating the immune response against invading pathogens. By mitigating their activity, corticosteroids curtail the immune system's capacity to combat infections effectively [54].

Employing corticosteroids in septic shock management involves navigating a fine equilibrium. On one side of the spectrum, they can aid in attenuating the inflammatory cascade characteristic of sepsis, thus preventing further harm to tissues. On the flip side, the immunosuppressive effect can potentially compromise the host's ability to manage and clear infections [55]. The impact of hydrocortisone, a representative corticosteroid, extends to adaptive immunity, a facet of the immune system responsible for antibody production and the establishment of immune memory. These effects assume particular significance in the context of infections [56].

Corticosteroids such as hydrocortisone can hamper the generation of antibodies crucial for recognizing and neutralizing pathogens. This impairment in antibody production may undermine the body's capacity to mount an effective humoral immune response against invading microorganisms [57]. Furthermore, the use of corticosteroids has the potential to disrupt the formation and maintenance of immune memory within the adaptive immune system. This disruption could, in turn, render the patient more susceptible to recurrent infections, as immune memory cells are integral for providing enduring protection against previously encountered pathogens [57].

Impact on the Hypothalamic-Pituitary-Adrenal (HPA) Axis

Feedback regulation: Corticosteroids, such as hydrocortisone, exert a potent negative feedback effect on the HPA axis. When exogenous corticosteroids are introduced into the body, they act as signals to the hypothalamus and pituitary gland to reduce the production of corticotropin-releasing hormone (CRH) and adrenocorticotropic hormone (ACTH), respectively. This regulatory mechanism is part of the body's attempt to maintain cortisol levels within a narrow physiological range [58].

HPA suppression: The negative feedback loop triggered by exogenous corticosteroids effectively puts the HPA axis "on hold." The reduced secretion of CRH and ACTH leads to diminished adrenal gland stimulation, which produces less endogenous cortisol. This suppression of the HPA axis is a desired effect when using corticosteroids to manage conditions like inflammation or autoimmune diseases, where excess cortisol production can be harmful [59].

Adrenal suppression: While the negative feedback mechanism is therapeutic in certain situations, prolonged or high-dose hydrocortisone therapy can have unintended consequences, notably adrenal suppression. This occurs when the adrenal glands become less responsive to endogenous signals to produce cortisol, potentially leading to adrenal insufficiency once corticosteroid treatment is discontinued [60].

Risk of adrenal insufficiency: Adrenal suppression can present a significant clinical concern, particularly in patients who have received corticosteroids over an extended period or at high doses. When hydrocortisone is abruptly discontinued or tapered too rapidly, the adrenal glands may not immediately resume normal cortisol production. This can result in relative adrenal insufficiency, where the body cannot mount an appropriate stress response, potentially leading to severe complications [61].

Controversies and debates

The Role of Other Corticosteroids

A notable point of contention in septic shock treatment revolves around whether hydrocortisone represents the optimal corticosteroid choice. Some studies have explored alternative corticosteroids, with dexamethasone being a prominent contender. The ongoing research agenda is focused on elucidating these alternatives' comparative efficacy, safety profiles, and appropriate dosing regimens [62]. During the COVID-19 pandemic, dexamethasone garnered considerable attention as a therapeutic option for managing severe respiratory distress. Its role in the treatment of septic shock, especially in the context of COVID-19, has piqued interest due to the shared pathophysiological characteristics between the two conditions [63].

Steroid-Resistant Patients

The concept of steroid resistance in the context of septic shock warrants precise delineation. The challenge lies in pinpointing which patients exhibit resistance to steroids and determining the appropriateness of alternative treatments or escalated corticosteroid dosage issues that spark debates within the medical community [15]. Comprehending the mechanisms that underlie steroid resistance in specific patients assumes paramount significance. Factors encompassing altered expression of glucocorticoid receptors, genetic polymorphisms, and disparities in the host's inflammatory response mechanisms may all contribute to the development of steroid resistance [64].

Impact on Secondary Infections

The immunosuppressive effects of corticosteroids introduce apprehension regarding an elevated susceptibility to secondary infections among patients grappling with septic shock. Striking a delicate equilibrium between reaping the anti-inflammatory advantages and navigating the potential infection risks constitutes a pivotal challenge in clinical decision-making [27]. Considerable debates revolve around formulating strategies to mitigate the jeopardy of secondary infections in this context. These strategies include implementing prophylactic antibiotics or vigilant monitoring for signs of infection during corticosteroid treatment [65].

Long-Term Consequences of Hydrocortisone Use

Extended or high-dose hydrocortisone therapy carries the potential to induce adrenal suppression, which may subsequently culminate in adrenal insufficiency upon treatment cessation. The enduring ramifications of adrenal suppression, encompassing the necessity for steroid replacement therapy and the possibility of adrenal recovery, are currently subjects under active investigation [66]. Moreover, emerging research suggests that corticosteroid therapy may exert cognitive and psychological effects, particularly when administered over prolonged durations. These effects encompass mood disturbances, cognitive deficits, and alterations in emotional well-being. However, these effects' full extent and clinical significance necessitate further investigation to provide a comprehensive understanding [67].

Clinical guidelines and recommendations

Current Guidelines from Medical Organizations

Surviving Sepsis Campaign: The Surviving Sepsis Campaign, an esteemed international collaboration, has played a pivotal role in shaping the landscape of septic shock management guidelines. This campaign provides comprehensive recommendations encompassing various facets of sepsis and septic shock care, including the administration of hydrocortisone. These guidelines serve as a foundational resource for healthcare providers in their pursuit of optimal patient outcomes [68].

Society of Critical Care Medicine (SCCM): The SCCM is a leading authority in critical care medicine. It offers clinical practice guidelines and authoritative position statements for septic shock management. These documents are rooted in a synthesis of rigorous evidence and expert consensus, providing invaluable guidance on diverse aspects of care for patients with septic shock [69].

Infectious Diseases Society of America (IDSA): The IDSA specializes in producing guidelines that often focus on specific dimensions of infectious diseases, including sepsis. These guidelines offer insights into the intricate management of septic shock, particularly in the context of distinct infectious agents. They are a valuable resource for healthcare professionals seeking nuanced approaches to septic shock care [70].

Interpretation and Application of Guidelines in Clinical Practice

Guideline updates: Guidelines are not static documents; they evolve with new evidence and research. Healthcare professionals must stay current with the latest updates to ensure they are implementing the most recent recommendations. Regularly reviewing and incorporating guideline revisions into practice is essential for providing evidence-based care [71].

Individualized patient care: While guidelines offer valuable guidance, clinical decision-making should always be tailored to each patient's unique circumstances. Healthcare providers should consider the patient's clinical presentation, comorbidities, response to treatment, and personal preferences. A patient-centered approach ensures that care aligns with the individual's needs and values.

Multidisciplinary approach: Managing complex conditions like septic shock often requires a multidisciplinary approach. Collaboration among intensivists, infectious disease specialists, pharmacists, and other healthcare providers is essential. Each team member brings valuable expertise, ensuring that guidelines are applied comprehensively and effectively.

Clinical judgment: In situations where guidelines do not offer clear-cut answers, healthcare professionals must exercise clinical judgment. This is especially relevant in cases with limited evidence or when patients present with complex conditions. Expertise and experience are critical in making informed decisions that prioritize patient well-being.

Shared decision-making: Informed and shared decision-making is a cornerstone of patient-centered care. Healthcare providers should engage in open and transparent discussions with patients and their families. These discussions should thoroughly explore the risks, benefits, and alternatives to treatments like hydrocortisone therapy. Patients should actively participate in care decisions, with their autonomy and values respected.

Quality improvement initiatives: Hospitals and healthcare institutions can leverage guidelines to implement quality improvement initiatives. These initiatives aim to standardize care processes, improving consistency and patient outcomes. Protocols for early recognition and management of conditions like septic shock can be developed and refined based on guidelines to enhance care delivery.

Education and training: Ensuring that healthcare providers are well-educated and trained in interpreting and applying guidelines is vital. This education should encompass the guidelines' content and practical implementation in clinical settings. Continuous learning and skill development empower healthcare professionals to integrate guidelines effectively into their practice.

Continuous evaluation: Regularly evaluating the outcomes of septic shock management is essential. This involves monitoring guideline adherence, assessing patient outcomes, and conducting quality improvement reviews. This ongoing evaluation allows healthcare institutions to identify areas for improvement, refine practices, and ultimately enhance the quality of care provided.

Practical considerations for hydrocortisone administration

Hospital Protocols and Procedures

Standardized protocols: Hospitals must develop and implement standardized protocols for administering hydrocortisone in septic shock. These protocols should be based on current clinical guidelines and best practices, considering the most up-to-date medical literature and expert recommendations. Standardization ensures all patients receive consistent, evidence-based care, improving treatment outcomes and patient safety.

Interdisciplinary collaboration: Effective communication and collaboration among healthcare teams are pivotal in delivering optimal care to patients with septic shock. Hospital protocols should clearly define the roles and responsibilities of various healthcare providers involved in the patient's care. This includes delineating the responsibilities of physicians, nurses, pharmacists, and laboratory staff. Interdisciplinary collaboration promotes efficient and coordinated care, reducing the risk of errors and enhancing patient outcomes.

Medication management: Hospitals should establish rigorous procedures for the safe and accurate preparation and administration of hydrocortisone. These procedures should encompass various aspects, including specifying dosing regimens based on patient characteristics and the severity of septic shock. In cases where dilution is necessary, clear and precise instructions should be provided. Additionally, hospitals should ensure compatibility assessments with other medications commonly administered to septic shock patients. These measures safeguard against medication errors and adverse drug interactions.

Documentation: Comprehensive documentation practices are fundamental to patient care and accountability. Hospitals should establish clear guidelines for documenting hydrocortisone administration, patient responses, and any adverse events or complications that may arise during treatment. Timely and accurate documentation is essential for tracking patient progress and ensuring accountability among healthcare providers. Proper documentation is critical for auditing and improving care processes and for legal and regulatory compliance.

Monitoring and Follow-Up

Hemodynamic monitoring: Continuous and meticulous monitoring of hemodynamic parameters is essential throughout hydrocortisone administration. This includes monitoring blood pressure, heart rate, and central venous pressure. Frequent assessments provide valuable insights into the patient's cardiovascular stability and guide adjustments to hydrocortisone therapy as needed. Hemodynamic monitoring aids in gauging the effectiveness of treatment and helps healthcare providers respond promptly to changes in the patient's condition.

Laboratory tests: Regular laboratory testing is a cornerstone of patient care during hydrocortisone therapy. This should encompass a range of assessments, including complete blood counts, electrolyte panels, and blood glucose measurements. These tests serve multiple purposes, including the early detection of potential complications such as hyperglycemia or electrolyte imbalances. Timely identifying these issues enables healthcare providers to implement appropriate interventions and mitigate adverse effects.

Clinical assessment: Beyond laboratory tests and hemodynamic parameters, clinical assessment remains critical to monitoring. Healthcare providers should closely monitor clinical signs indicating the resolution of septic shock. These signs may include improved tissue perfusion, reduced vasopressor support requirements, and organ dysfunction resolution. Regular clinical assessments enable healthcare providers to decide whether to continue or modify hydrocortisone therapy.

Adverse effects: Vigilance for potential adverse effects associated with hydrocortisone therapy is imperative. These may include hyperglycemia, an increased risk of secondary infections, or psychological effects. Healthcare providers must be prepared to address any adverse events that may arise during treatment promptly. Proactive management of adverse effects contributes to patient safety and enhances the overall quality of care.

Response to treatment: Continuous evaluation of the patient's response to hydrocortisone therapy is essential. If there is a lack of improvement or a worsening of the patient's condition despite treatment, healthcare providers should consider alternative management strategies. This may involve reevaluating the diagnosis, reassessing the underlying cause of septic shock, or exploring other therapeutic options in consultation with specialists.

Patient and Family Education

Patient care and treatment involve a collaborative effort between healthcare providers and patients, and patients or their legally authorized representatives must be fully informed and engaged in the decision-making process. One crucial aspect of this is obtaining informed consent before initiating hydrocortisone therapy. This process entails a comprehensive discussion with patients or their representatives, during which healthcare professionals explain the purpose, potential benefits, risks, and available treatment alternatives of hydrocortisone therapy. To ensure that patients and their families thoroughly understand hydrocortisone therapy, healthcare providers must provide clear and accessible information. This includes explaining how hydrocortisone works, its intended effects on the body, and the expected course of treatment. It is paramount to ensure that this information is communicated in a language and format that patients and their families can comprehend.

Hydrocortisone therapy may be accompanied by potential adverse effects that patients and their families should be aware of. Healthcare providers should educate them about these potential side effects and the necessary monitoring requirements during treatment. Emphasizing the importance of regular follow-up appointments and laboratory tests is vital to ensure that any adverse effects are detected and managed promptly. In cases where hydrocortisone therapy extends beyond the hospital setting, such as in-home care, educating patients and their families on the dosing regimen and administration techniques becomes essential. Clear and concise instructions must be provided to ensure the therapy is administered correctly and consistently, minimizing the risk of errors.

Empowering patients and their families to participate in their care actively is fundamental to patient education. They should be encouraged to promptly report any concerning symptoms, changes in condition, or medication-related issues to their healthcare providers. This proactive approach fosters better communication and enhances patient safety. Recognizing the psychological impact of septic shock and its treatment on patients and their families is equally important. Healthcare providers should be attuned to the emotional challenges that may arise and offer appropriate emotional support. Counseling services should also be available when needed to address any psychological distress. To facilitate ongoing understanding and reference, healthcare providers should provide written materials or resources that patients and their families can access for additional information and clarification. These materials can serve as valuable references, ensuring that patients and their families are well-informed throughout hydrocortisone therapy. Patient and family education is a cornerstone of effective healthcare, promoting informed decision-making, patient engagement, and optimal treatment outcomes.

Future directions and research opportunities

Emerging Therapies and Adjunctive Treatments

In septic shock management, ongoing research is dedicated to exploring the potential of innovative immunomodulatory agents as tools for fine-tuning the immune response. This includes in-depth investigations into applying monoclonal antibodies, cytokine inhibitors, and toll-like receptor (TLR) agonists as adjunctive treatments to enhance treatment effectiveness. The role of the gut microbiome in the context of septic shock is garnering increasing attention within the scientific community. Subsequent research endeavors may delve into interventions specifically targeting the microbiome, potentially leading to the modulation of host immune responses and ultimately resulting in improved patient outcomes. Furthermore, a promising avenue of inquiry lies in cell-based therapies, notably mesenchymal stem cell (MSC) treatments, celebrated for their immunomodulatory properties and tissue-regenerative potential. The ongoing exploration of the safety and efficacy of these therapies in the context of septic shock has sparked significant interest within the medical community.

Precision Medicine Approaches

Precision medicine strategies in septic shock hinge upon identifying genetic markers that could influence patient responses to treatment. Genomic profiling, in particular, is poised to play a pivotal role in tailoring therapies to the unique characteristics of individual patients, thereby optimizing the likelihood of achieving positive treatment outcomes. Beyond the scope of genomics, characterizing patient phenotypes based on their distinctive clinical and biomarker profiles can facilitate more precise and individualized treatment strategies. These phenotypic classifications can guide the selection of specific therapies or interventions that align closely with each patient's needs.

Biomarkers for Patient Stratification

Identifying biomarkers capable of predicting disease severity, treatment response, or the risk of complications carries profound implications for clinical decision-making. Future research endeavors are poised to uncover novel biomarkers that enable the stratification of patients based on their specific clinical characteristics and risk profiles. Predictive biomarkers are particularly interesting; they can forecast a patient's likelihood of responding favorably to hydrocortisone or other therapeutic interventions. This could prove instrumental in avoiding unnecessary treatments and their associated risks, thereby enhancing the overall efficiency of septic shock management.

Targeted Therapies Based on Pathophysiological Subtypes

Recognizing the inherent heterogeneity of septic shock as a condition, future research is poised to pivot towards subphenotyping patients based on the underlying pathophysiological mechanisms that drive their disease. This innovative approach could pave the way for the development of tailored therapies that are finely attuned to address the specific requirements of each subgroup. Ultimately, the objective is to craft personalized treatment plans for septic shock patients, informed by their subphenotype and individual characteristics. This could entail a synergy of corticosteroids with other therapies or interventions meticulously tailored to align with the patient's unique pathophysiological profile.

## Conclusions

In conclusion, the management of septic shock poses a nuanced and dynamic challenge in critical care medicine. This comprehensive review delved into historical perspectives, current evidence, controversies, mechanisms of action, and practical considerations related to hydrocortisone administration in septic shock. The multifaceted role of hydrocortisone as an adjunctive therapy in septic shock is evident, demonstrating potential benefits in stabilizing hemodynamics, mitigating the inflammatory response, and improving patient outcomes. However, its application is not without controversies, and uncertainties persist regarding patient selection, optimal dosing regimens, safety profiles, and long-term consequences. The future trajectory of septic shock management hinges on pursuing precision medicine, exploring emerging therapies, discovering relevant biomarkers, and implementing targeted interventions based on distinct pathophysiological subtypes. While clinical guidelines offer a valuable framework, the crux of effective treatment lies in individualized patient care, fostering interdisciplinary collaboration, and perpetuating ongoing research. Moving forward, a resounding call for continued research and an unwavering commitment to evidence-based practice underscores our dedication to enhancing the care of septic shock patients and advancing outcomes in this critical medical condition.
